# Alfalfa Photosynthesis Under Partial Root-Zone Drying: Diurnal Patterns and Its Non-Stomatal Limitations

**DOI:** 10.3390/plants14111573

**Published:** 2025-05-22

**Authors:** Yadong Wang, Qiuchi Zhang, Mingxiu Ju, Kai Gao, Liliang Han, Xingfu Li, Jing He, Derong Su

**Affiliations:** 1 College of Grassland Science, Inner Mongolia Minzu University, Tongliao 028000, China; wangyadong@imun.edu.cn (Y.W.); 18648524152@163.com (Q.Z.); jumingxiu2024@imun.edu.cn (M.J.); 2 Academy of Forestry Inventory and Planning, National Forestry and Grassland Administration of P.R. China, Beijing 100714, China; hanliliang1024@126.com; 3 Industry Development and Planning Institute, National Forestry and Grassland Administration of P.R. China, Beijing 100010, China; lixingfu0@163.com; 4 College of Grassland Science, Beijing Forestry University, Beijing 100083, China; hejing_606@163.com

**Keywords:** alternate partial root-zone irrigation, lucerne, water stress, water-use efficiency

## Abstract

The effects of stomatal factors of plant leaves under partial root-zone drying (PRD) have been widely studied. However, the non-stomatal factors and the relationship between photosynthesis with soil moisture have not been explored. In this study, four treatments over-irrigation, full irrigation, moderate water deficit, and severe water deficit were investigated, aiming to evaluate the effects on the diurnal variation of alfalfa leaf photosynthesis under PRD and its relationship with stomatal and non-stomatal limitations, as well as soil moisture. The results showed that any levels of water deficit led to a decrease in the photosynthetic rate (*P_n_*) of alfalfa leaves. Leaves under moderate and severe water deficit displayed a pronounced midday “photosynthetic lunch break,” while those under over- and full irrigation did not display this phenomenon. Before 11:30 a.m., the reduction in *P_n_* was primarily due to stomatal limitations, as evidenced by reduced stomatal conductance (*G_s_*) and decreased intercellular CO_2_ concentration (*C_i_*). After 11:30 a.m., non-stomatal limitations became the dominant factor, with both *G_s_* and transpiration rate (*T_r_*) continuing to decrease, while *C_i_* increased, indicating a shift in the limiting factors. Under PRD with moderate water deficit, alfalfa experienced both stomatal and non-stomatal limitations within a single day, leading to a hay yield reduction of 18.6%. Additionally, over-irrigation helped to maintain higher *P_n_* and *T_r_*, increasing alfalfa yield and thus improving water productivity by 33.1%. The correlation coefficients between soil moisture content at 10 cm depths with alfalfa leaf *P_n_*, *T_r_*, and *G_s_* on the photosynthetic measurement day were 0.9864, 0.8571, and 0.8462, respectively. At 20 cm, the correlation coefficients were 0.8820, 0.6943, and 0.6951, respectively. The study concluded that both stomatal and non-stomatal mechanisms contributed to reduced alfalfa *P_n_* in water deficit of PRD. Furthermore, shallow soil moisture also played a crucial role in influencing photosynthetic performance.

## 1. Introduction

Partial root-zone drying (PRD) has been widely studied for its role in regulating plant responses to soil drought, primarily through the action of abscisic acid (ABA) [1,2]. In early PRD studies on fruit trees, Davies et al. [3] discovered that ABA was released from the dry side of the root system, leading to stomatal closure in the leaves—a physiological basis for PRD [4,5]. This results in reduced stomatal conductance (*G_s_*) and a decreased influx of CO_2_, leading to a limitation in photosynthesis, known as stomatal limitation [6,7]. 

A general consensus exists in the literature that PRD improves the leaf intrinsic water-use efficiency (*WUE_i_*). However, there is some disagreement about the mechanisms behind this improvement. Some studies suggest that PRD increases *WUE_i_* by significantly reducing *G_s_* without affecting the photosynthetic rate (*P_n_*) [8,9,10,11]. On the other hand, some studies report that PRD can increase *P_n_* [12,13], while others suggest that PRD reduces *P_n_*, though not to the extent of the reduction in *G_s_* [14,15]. These conflicting results create uncertainty in our understanding of the full impact of PRD on plant photosynthesis.

The underlying photosynthetic mechanisms of PRD under varying levels of over-irrigation and water stress are still not fully understood. Stomatal behavior in PRD is regulated by chemical signals that convey information about water availability to the shoot [4,5]. A key signaling component is the plant hormone ABA, which is produced in both the roots and shoots and transported to the leaves, where it triggers stomatal closure [3,4]. In addition to stomatal regulation, non-stomatal factors play a significant role in regulating *WUE_i_* under water-stress conditions [16,17]. These factors—including increased leaf temperature, reduced chloroplast activity, and suppressed Rubisco activity—all contribute to decreased photosynthetic capacity, referred to as non-stomatal limitations of photosynthesis [5]. Furthermore, the impact of over-irrigation on photosynthetic characteristics under PRD—particularly whether it leads to a decrease in *P_n_* [18]—and the relationship with diurnal variation of photosynthesis, remain largely unexplored. There is still a considerable gap in our understanding of the responses of non-stomatal factors to both over-irrigation and water stress under PRD.

Moreover, over-irrigation and water deficit under PRD also impact soil moisture [19,20], which has an indirect effect on photosynthetic intensity [21,22,23,24]. However, the relationship between photosynthesis and soil moisture under both over-irrigation and water stresses in PRD has been insufficiently explored. In this study, we used alfalfa as a model to investigate photosynthetic characteristics under over-irrigation, full irrigation, moderate water deficit, and severe water-deficit conditions following two cycles of PRD. We hypothesized that water deficit under PRD conditions would reduce the *G_s_* lower than *P_n_* of alfalfa to increase *WUE_i_*. Further, we believed that not only stomatal factors played a role, but non-stomatal factors were likely to play an important role. In addition, soil moisture might have a significant relationship with photosynthetic indicators. The results help us to understand the mechanism by which PRD improves *WUE_i_*.

## 2. Results 

### 2.1. Meteorological Data

**Figure 1:**
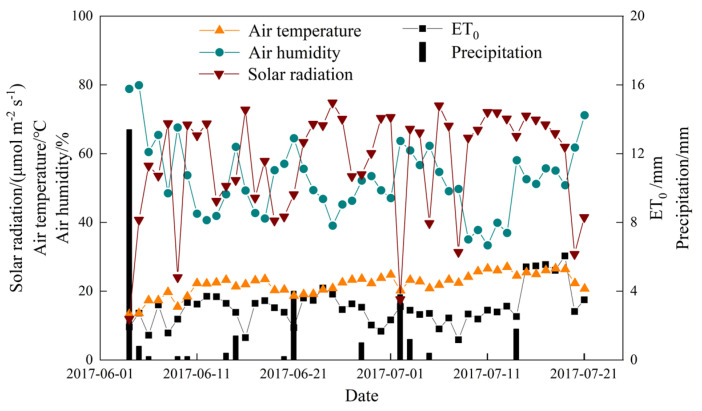
Meteorological changes during the experiment.

Air temperature, humidity, solar radiation, and precipitation during the experiment, along with the reference crop evapotranspiration (ET0) as outlined by Allen et al. [25], are presented in Figure 1.

**Figure 2:**
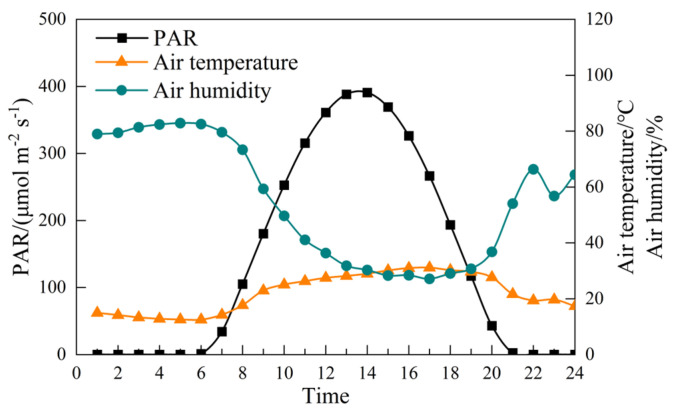
Meteorological data on July 6, 2017. PAR: photosynthetically active radiation.

During the first alfalfa growing season in 2017, a total of 13.4 mm of rainfall was recorded (Table 1). However, the average daily rainfall during the experiment did not meet the effective rainfall threshold of 5 mm (Figure 1). The meteorological conditions for 6 July 2017, are presented in Figure 2. On this day, photosynthetically active radiation (PAR) and air temperature showed an initial increase, followed by a decrease over the course of the day, with time series data reflecting this pattern. In contrast, air humidity exhibited an inverse trend, decreasing as temperature and PAR increased. The maximum PAR reached 390.9 μmol m^−2^ s^−1^ at 14:00, while the air temperature peaked at 31.13 °C at 17:00. The air humidity was lowest at 27.15% at this point in time. 

### 2.2. Photosynthesis

**Figure 3:**
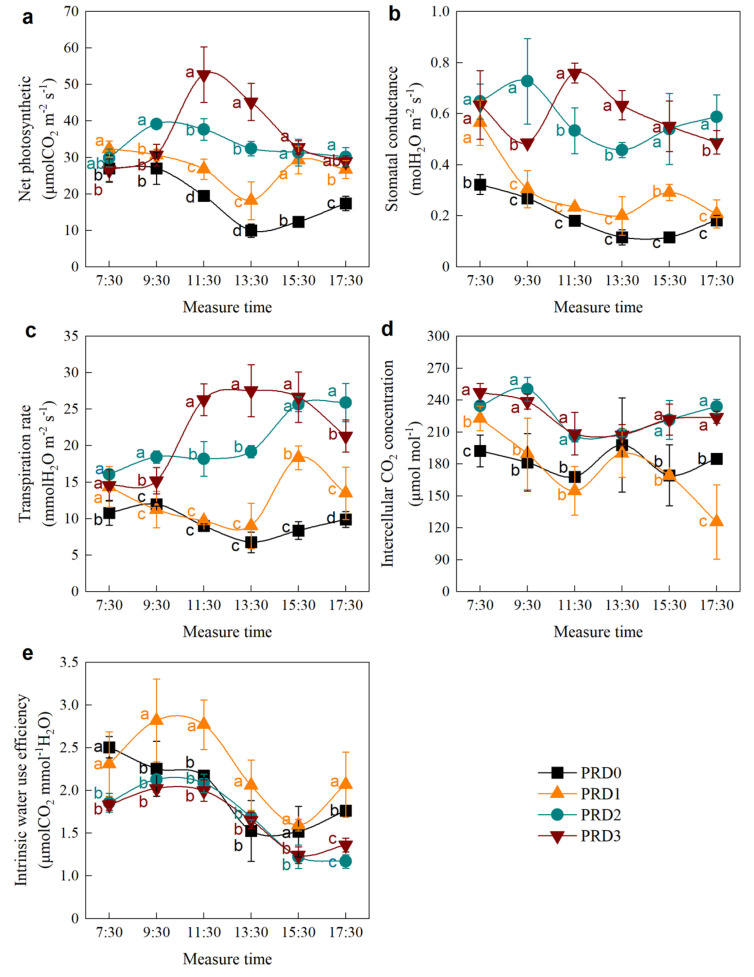
Diurnal changes of net photosynthetic (**a**), stomatal conductance (**b**), transpiration rate (**c**), intercellular CO_2_ concentration (**d**), and intrinsic water-use efficiency (**e**) of alfalfa leaves on 6 July 2017. Different lowercase letters indicate differences between treatments at the same measurement time.

Figure 3 illustrates the daily changes in alfalfa photosynthesis following two rounds of PRD. As shown in Figure 3a, the *P_n_* under the PRD3 treatment followed a “single peak” curve, with the maximum *P_n_* occurring at 11:30 a.m. (52.70 μmol m⁻^2^ s⁻^1^). In contrast, the *P_n_* of the PRD2 treatment exhibited a weaker “single peak” curve, reaching its maximum at 9:30 a.m. (39.17 μmol m⁻^2^ s⁻^1^). The moderate-water-deficit PRD1 treatments displayed “double peak” curves. The PRD1 treatment showed two peaks at 7:30 a.m. and 15:30 p.m., with the second peak being weaker than the first. 

In contrast to *P_n_*, the daily variation in *G_s_* of alfalfa under PRD treatments exhibited a “double peak” pattern across all irrigation treatments (Figure 3b). Specifically, the first peak in PRD3 was lower than the second peak, while for PRD2, PRD1, and PRD0, the first peak of *G_s_* was higher than the second. Except at 7:30 a.m., *G_s_* in the PRD3 and PRD2 treatments were significantly higher than those in the PRD1 and PRD0 treatments, suggesting that water deficit under PRD conditions reduced leaf stomatal conductance.

Unlike *P_n_* and *G_s_*, the *T_r_* in the PRD3 treatment remained relatively high from 11:30 to 15:30, with the peak value of 27.54 mmol m⁻^2^ s⁻^1^ observed at 13:30 p.m. In the PRD2 treatment, *T_r_* increased progressively with time, reaching its maximum at 17:30 p.m. The *T_r_* in PRD2 followed a “double peak” pattern, with the second peak higher than the first. In the PRD1 treatment, *T_r_* initially increased and then decreased with time, reaching its lowest point at 13:30 p.m. before peaking again at 15:30 p.m.

*C_i_* exhibited similar trends for high irrigation treatments (PRD3 and PRD2) and low irrigation treatments (PRD1 and PRD0), with some differences observed at specific time points (7:30 a.m. and 17:30 p.m.). At 7:30 a.m., *C_i_* in all PRD treatments was significantly higher than in the non-irrigated PRD0 treatment (Figure 3d) (*p* < 0.05). Additionally, except at 13:30 p.m., *C_i_* in the over-irrigation treatment PRD3 and the full irrigation treatment PRD2 were significantly higher than in the water-deficit treatments (PRD1 and PRD0) (*p* < 0.05).

Under PRD conditions, the *WUE_i_* of alfalfa leaves showed a “double peak” curve (Figure 3e). Notably, the first peak of *WUE_i_* in all irrigation treatment was higher than the second peak. *WUE_i_* reached its peak at 9:30 a.m. and reached its bottom at 15:30 p.m. Furthermore, the *WUE_i_* of the moderate-water-deficit PRD1 treatment was higher than that of the PRD2 and PRD3.

**Figure 4:**
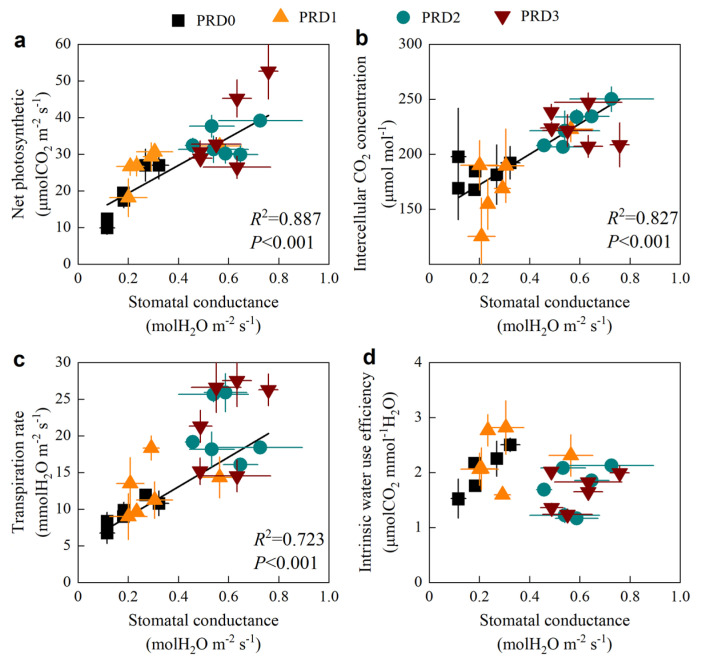
Relationship between stomatal conductance with net photosynthetic (**a**), intercellular CO_2_ concentration (**b**), transpiration rate (**c**), and intrinsic water-use efficiency (**d**).

The relationship between *G_s_* and various photosynthetic physiological indicators is shown in Figure 4. The linear relationships between *G_s_* and *P_n_*, *C_i_* and *T_r_* are the following: y = 37.88x + 11.84 (R^2^ = 0.887), y = 138.87x + 144.57 (R^2^ = 0.827), and y = 20.21x + 4.98 (R^2^ = 0.723), respectively.

### 2.3. Stomatal and Non-Stomatal Limitation

**Figure 5:**
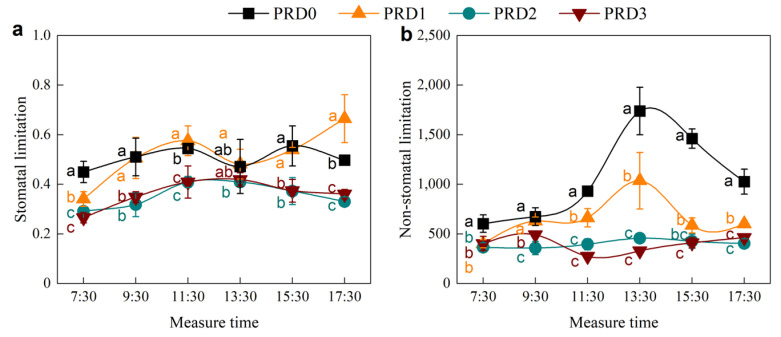
Diurnal variation of alfalfa stomatal (**a**) and non-stomatal limits (**b**) under PRD. Different lowercase letters indicate differences between treatments at the same measurement time.

Figure 5 presents the daily changes in *L_s_* (Figure 5a) and non-stomatal limitation (Figure 5b) of alfalfa under different PRD treatments. The daily changes of *L_s_* for the PRD3 and PRD2 treatments followed a “single peak” curve, while the PRD1 and PRD0 treatments exhibited a “double peak” curve. With the exception of 13:30 p.m., *L_s_* values in the PRD1 and PRD0 treatments were higher than those in the PRD3 and PRD2 treatments, indicating that stomatal limitation in alfalfa leaves was more pronounced under PRD-induced water-deficit conditions. There were no significant differences among the treatments at 13:30 p.m. (*p* > 0.05), where stomatal limitation reached its maximum under both excessive and sufficient irrigation. At this point, stomatal limitation was smaller under the PRD water-deficit conditions.

The daily variation of non-stomatal limitation value of alfalfa under PRD was significantly lower in the treatments of over-irrigation PRD3 and full irrigation PRD2 compared to the deficit irrigation treatments (PRD1 and PRD0) (*p* < 0.05). Non-stomatal limitation in the PRD1 and PRD0 treatments followed a “single peak” pattern, peaking at 13:30 p.m., with values of 1036.42 and 1738.89, respectively. Compared to PRD0, non-stomatal limitations in PRD1, PRD2, and PRD3 decreased by 40.40%, 73.71%, and 71.91% at 13:30 p.m., respectively. At this time, non-stomatal limitation occurred in PRD0 and PRD1. 

**Figure 6:**
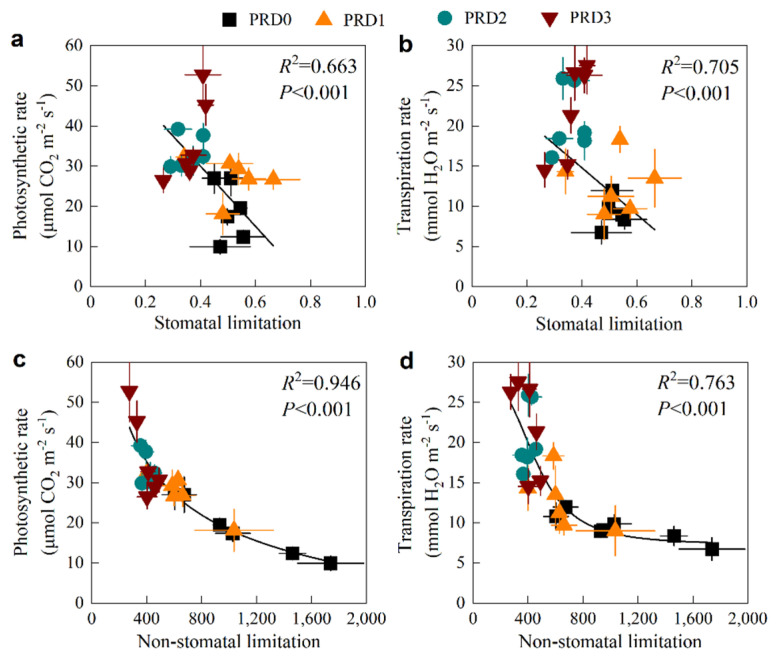
Relationship between stomatal and non-stomatal limits with photosynthetic rate (**a**,**c**) and transpiration rate (**b**,**d**).

The relationship between *L_s_* and non-stomatal limitation value and *P_n_* and *T_r_* is shown in Figure 6. The relationship between *L_s_* with *P_n_* and *T_r_* is y = 59.95 − 74.86x (R^2^ = 0.663) and y = 26.43 − 29.09x (R^2^ = 0.705), respectively. The relationship between the non-stomatal limit value with *P_n_* and *T_r_* is a logistics curve, which is y = 401.29/(1 + x/6.47) − 22.63 (R^2^ = 0.946) and y = 8.18/(1 + x/600.53) + 9.70 (R^2^ = 0.763), respectively. In addition, we observed a highly significant positive correlation between the *C_i_* and the *P_n_* in y = 0.22x − 18.58 (R^2^ = 0.672) (Supplementary Figure S1).

### 2.4. Soil Moisture

**Figure 7:**
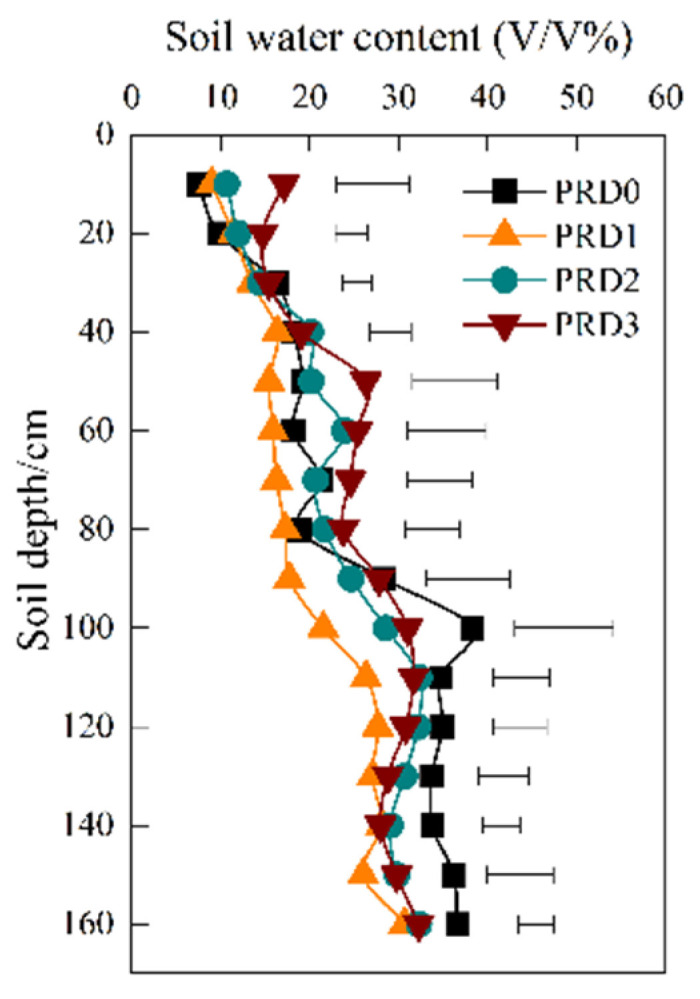
Soil moisture of 10~160 cm layer on photosynthetic measurement day.

On 6 July 2017, soil moisture in the shallow 10 cm and 20 cm layers showed an increasing trend with the increase in irrigation (Figure 7). Compared to the PRD0 treatment, soil moisture at 10 cm in the PRD1, PRD2, and PRD3 treatments increased by 19.82%, 42.28%, and 128.43%, respectively. At 20 cm, the increases were 16.57%, 22.57%, and 50.50%, respectively. Figure 7 indicates that, starting from the 30 cm soil layer, soil moisture in the PRD1 treatment decreased to varying extents compared to the PRD0 treatment. The smallest decrease occurred in the 60 cm soil layer (7.36%), while the largest decrease was observed in the 100 cm layer (43.74%). In comparison to PRD0, except for the 60 cm soil layer, where moisture increased, soil moisture in all other layers decreased in the PRD2 treatment. In the PRD3 treatment, soil moisture increased in the 10–20 and 50–80 cm range, while it decreased in other layers.

### 2.5. Relationship Between Soil Moisture and Photosynthetic Parameters

The regression expression describing the relationship between soil moisture and photosynthetic parameters at 10 and 20 cm soil layers on July 6, 2017 was the following: y = y0 + A × exp(R0 × x) (Table 2). The determination coefficients of the relationship between soil moisture at 10 cm layer with *P_n_*, *T_r_*, and *G_s_* were 0.9864, 0.8571, and 0.8462, respectively. For the 20 cm layer, the determination coefficients for the relationships with *P_n_*, *T_r_*, and *G_s_* were 0.8820, 0.6943, and 0.6951, respectively. 

### 2.6. Hay Yield and Water Productivity

Table 3 summarizes the effects of different PRD treatments on alfalfa hay yield. The hay yield followed the order PRD3 > PRD2 > PRD1 > PRD0, with extremely significant differences observed among the treatments (*p* < 0.01). The yield of the over-irrigation PRD3 treatment was 1.17 times higher than that of the severe water-deficit PRD0 treatment, indicating a significant decline in alfalfa hay yield as the severity of the PRD water deficit increased.

The WP of the PRD3 treatment was significantly higher than that of the other treatments. Specifically, the WP of PRD0, PRD1, and PRD2 treatments decreased by 25.21%, 31.54%, and 24.89%, respectively. PRD3 achieved a higher WP by increasing hay yield (Table 2) while reducing soil moisture (Figure 5). The irrigation water productivity (IWP) of the PRD0 treatment was significantly higher than that of the other treatments (*p* < 0.01). Additionally, the IWP of PRD0 and PRD1 treatments was significantly higher than that of PRD2 and PRD3 (*p* < 0.05).

## 3. Discussion

Unlike traditional irrigation, which uniformly wets the active root zone, PRD alternates between wet and dry portions of the root zone. In the dry area, water deficit triggers the roots to release ABA, which is transported to the leaves through transpiration, causing stomatal closure and reducing leaf *G_s_* [4,5,26]. Since the sensitivity of *P_n_* to PRD is less pronounced than that of *G_s_*, this results in an increase in *WUE_i_* [5,8,18,26]. As we predicted before, under moderate-water-deficit PRD (PRD1), the decrease in leaf *G_s_* was greater than the decrease in *P_n_* (Figure 3a,b), thereby increasing the leaf *WUE_i_* (Figure 3e). However, this phenomenon was not observed under full or over-irrigation (Figure 3e), indicating that water deficit, rather than PRD itself, is the main factor responsible for the increase in *WUE_i_*.

Furthermore, moderate water deficit under PRD caused a significant reduction in alfalfa *P_n_* (Figure 3a), which aligns with the findings of Zhang et al. [18]. Although we did not see any discussion on the effect of water-deficit PRD on photosynthesis in their research [18], we can get some insights from the previous study on the effect of drought stress on alfalfa photosynthesis. Erice et al. [27] and Aranjuelo et al. [28,29,30] found that water deficit did not reduce alfalfa leaf *P_n_*. The discrepancy may arise from their water-deficit conditions (typically at 70% field capacity), which may not have been severe enough to trigger a significant reduction in *P_n_*. This highlights the strong ability of perennial alfalfa to avoid dehydration [31,32]. Alfalfa plants tend to minimize water loss through effective stomatal control or by reducing light absorption [33], and this dehydration avoidance may also involve slowing the growth of new shoots to reduce water loss [34]. 

However, the maximum *P_n_* of alfalfa under our PRD3 can reach 52.70 μmol m⁻^2^ s⁻^1^, which is higher than most studies [18,27,28,29,30]. No other study compared the PRD and found that alfalfa could achieve such a high *P_n_* of alfalfa under excessive irrigation at noon. On the other hand, the temperature and light radiation at the research site have reached the highest at noon, and excessive irrigation makes alfalfa have no “photosynthetic lunch break” phenomenon at this time. Alfalfa leaves subjected to full irrigation under PRD also did not show the “photosynthetic lunch break” phenomenon, whereas those under moderate water deficit exhibited a clear midday reduction in photosynthesis (Figure 1). Du et al. [19] and Romero et al., [35] observed similar “lunch break” phenomena in cotton and grapevine under PRD with reduced irrigation. The photosynthetic “lunch break” caused by water deficit has been observed in a variety of plants [26,36], but whether this phenomenon behaves differently under PRD conditions remains to be further explored.

Both stomatal and non-stomatal limitations occurred within a single day, particularly under moderate-water-deficit PRD, as we expected (Figure 5). Before 11:30 a.m., the reduction in *P_n_* was primarily attributed to stomatal limitations (Figure 5a). Under water-deficit conditions, the stomatal resistance of alfalfa leaves increased or even completely closed. As a result, *G_s_* decreased, reducing the amount of CO_2_ available at the carboxylation sites of mesophyll cells, which led to a decrease in *C_i_*. This phenomenon is characterized by a joint decrease in *G_s_* and *C_i_* (Figure 1), indicating stomatal limitation (Figure 5a). After 11:30 a.m., as air temperature approached 31 °C and photosynthetically active radiation increased to 390.9 μmol m^−2^ s^−1^ (Figure 2), non-stomatal limitations began to dominate (Figure 5b). This shift was likely due to insufficient RuBP regeneration in the leaves or a decrease in the activity of the Rubisco enzyme system [27,28,37]. Specifically, while *G_s_* and *T_r_* continued to decline, *C_i_* increased instead of decreasing (Figure 3b,c,d and Supplementary Figure S1), indicating a non-stomatal limitation (Figure 5b). After 15:30 p.m., moderate water stress shifted from non-stomatal limitation to stomatal limitation (Figure 5a). Similar findings have been reported in other studies examining water-deficit effects on photosynthesis [16,38].

Consistent with previous studies [39,40], our results show that water deficit under PRD significantly reduces hay yield, thus improving alfalfa WP (Table 2). Our research team has also observed similar effects of water deficit under subsurface drip irrigation [41,42] and sprinkler irrigation [43], where water deficit consistently reduced alfalfa yield. In comparison to over-irrigation (PRD3), the WP in treatments with moderate water deficit (PRD0, PRD1, and PRD2) decreased by approximately 24.89–31.54%. Interestingly, over-irrigation improved WP by increasing hay yield (Table 2). This observation aligns with findings in cotton [19]. However, other studies involving PRD with furrow irrigation found that over-irrigation (115% ET_0_) reduced alfalfa yield [44], and subsequent research confirmed this result [45,46,47,48]. The reasons behind these inconsistent findings warrant further investigation.

## 4. Materials and Methods

### 4.1. Experimental Location

The field experiment was conducted at the National Field Scientific Observation and Research Station of Wuwei Oasis Agricultural Ecosystem, located in Gansu Province (102°50′ E, 37°52′ N, 1580 m above sea level) (Supplementary Figure S2a) The station operates in a typical continental temperate arid climate. The average annual sunshine duration exceeds 3000 h, with an average temperature of 8 °C and an annual accumulated temperature (above 0 °C) of 3350 °C. The region receives an average annual evaporation of 2000 mm from open-water surfaces and an average multi-year precipitation of 164 mm. This area is a representative oasis irrigation agricultural region. The soil type is sandy loam, with an average soil bulk density of 1.48 g cm^−3^ in the 0–1.6 m soil layer. The field water holding capacity is 0.35 cm^3^ cm^−3^, and the permanent wilting point is 0.09 cm^3^ cm^−3^. Throughout the experiment, net radiation, air temperature, relative humidity, wind speed, and precipitation at an altitude of 2 m were automatically recorded by a meteorological monitoring system (HOBO, Campbell Scientific Inc., North Logan, UT, USA). The meteorological station was located approximately 20 m from the experimental site. 

### 4.2. Subsurface Drip Irrigation Design for Partial Root-Zone Drying

The alfalfa field drip irrigation system consists of a water pump intake device, a main pipe, a branch pipe, a capillary tube (PE drip irrigation, 16 mm diameter), and drippers (wall thickness: 0.4 mm; dripper spacing: 30 cm; dripper flow rate: 3 L/h). The capillary tubes are buried 30 cm underground. To implement alternating root-zone drying, two sets of drip irrigation systems were installed based on previous studies (Supplementary Figure S2c,d) [40,41]. The layout includes one tube and four rows of capillary tubes, which was modified to one tube with two rows and a row spacing of 10 cm. A water meter was installed in the experimental plot to monitor and control irrigation amounts, with two switch valves installed for control. The two subsurface drip irrigation systems were used to alternate irrigation, with the A system supplying irrigations 1, 3, and 5, and the B system supplying irrigations 2 and 4 (Supplementary Figure S2c,d).

### 4.3. Experimental Design

The experimental reference crop evapotranspiration (ET_0_) was calculated according to the guidelines provided by Allen et al. [25]. The cumulative average ET_0_ between two irrigations was 22.44 mm. The specific irrigation regime is shown in Table 1, with irrigation occurring weekly. Each treatment was replicated three times, with 12 experimental plots in total. The distribution of plots is shown in Supplementary Figure S1b. Each plot measured 6 m in length and 4 m in width, with 20 alfalfa planting rows per plot. The irrigation experiment commenced on 17 June 2017, and was followed by the first mowing on 18 July 2017. Table 1 shows the irrigation system of the first PRD crop of alfalfa in 2017. 

### 4.4. Site Management

The experimental plot was used for alfalfa production from 2012 to 2016 [44]. In early 2017, the area was plowed, wheeled, and harrowed to remove the taproot and developing root crown of alfalfa, ensuring uniformity across the experimental plot. Alfalfa (cv. 4020MF) was planted on 20 May 2017, with a seeding rate of 20 kg ha^−1^. The seeds were manually sown in drills with a sowing depth of 0.05 m and row spacing of 0.2 m. During the seedling stage, micro-sprinkler irrigation was applied with an irrigation quota of 10 mm once a week on 24 May, 28 May, 31 Ma, 8 June, and 12 June 2017 (Table 3). No irrigation was applied on 4 June 2017, due to rainfall of 13.4 mm. The experimental growing season spanned from May to July in 2017. Field management activities such as weeding during the alfalfa seedling stage were consistent across all treatments.

### 4.5. Measurements

#### 4.5.1. Soil Moisture

The soil moisture content of each plot was monitored every 2–3 days using a 10 cm vertical soil layer at a depth of 160 cm using the Diviner 2000 system (Sentek Pty Ltd., Australia). Two soil moisture measuring tubes were placed in each plot [40,41]. In addition, the gravimetric soil moisture was measured by the oven drying method and calibrated using the data from the Diviner 2000 system during the harvest stage. 

Soil water storage [49] was calculated according to formula (1):

(1) $$SWS=h\times \theta_{v}\times 10^{-1}$$

where SWS is soil water storage (mm); h is soil depth (cm); *θ_v_* is soil volume content (%). 

#### 4.5.2. Alfalfa Photosynthetic Characteristics

Photosynthetic characteristics of alfalfa’s top leaves just opened were measured at the end of branching (July 6, 2017) from 7:30 to 17:30 using a portable photosynthetic instrument (Li-6400XT, LI-COR, Inc., Lincoln, Nebraska, USA) under natural light conditions, including photosynthetic rate (*P_n_*, μmol m^−2^ s^−1^), transpiration rate (*T_r_*, mmol m^−2^ s^−1^), stomatal conductance (*G_s_,* mol m^−2^ s^−1^), and intercellular CO_2_ concentration (*C_i_*, μmol·mol^−1^). Leaf temperature, CO_2_ concentration, vapor pressure deficit were 30 °C, 400 ppm, and 3 kPa, respectively. Each measurement was repeated 4 times. Instantaneous water-use efficiency (*WUE_i_*, μmol mmol) is the ratio of *P_n_* to *G_s_*.

The stomatal limitation (*Ls*) [50] was calculated according to formula (2):

(2) $$Ls=1-\frac{C_{i}}{C_{a}}$$

where *C_a_* represents the atmospheric CO_2_ concentration (μmol·mol^−1^).

The non-stomatal limitation value is calculated by *C_i_*/*G_s_* [51].

#### 4.5.3. Hay Yield and Water Productivity

Hay yield (kg hm^−2^) was measured at the beginning of flowering with three replicates for each treatment [40,41]. 

Actual evapotranspiration (ET_a_, mm) is estimated using the water balance [40] Formula (3):

(3) $$ET_{a}=I+P+S-\Delta SWS-R-D$$

where, I—irrigation volume, mm; P—precipitation, mm; S—groundwater recharge, mm; ΔSWS—change in soil water storage in the 0–1.6 m soil layer, mm; R—surface runoff, mm; D—deep seepage, mm. In this experiment, since no surface runoff is generated under drip irrigation conditions—and the designed single irrigation volume is small and insufficient to form deep seepage—S, R, and D are ignored.

Alfalfa water productivity (WP, kg hm^−2^ mm^−1^) [52] was calculated according to Formula (4):

(4) $$\mathrm{WP}=\frac{\mathrm{Hay}\;\mathrm{yield}}{{\mathrm{ET}}_{a}}$$

Irrigation water productivity (WP_I_, kg hm^−2^ mm^−1^) was calculated according to Formula (5):

(5) $${\mathrm{WP}}_{I}=\frac{\mathrm{Hay}\;\mathrm{yield}}{\mathrm{IWU}}$$

where IWU represents the irrigation water applied during the growth period (mm).

### 4.6. Statistical Analysis

The significance of the differences between the mean values of each treatment was analyzed by one-way analysis of variance (ANOVA) using IBM SPSS Version 20 (IBM Corp., Armonk, NY, USA). Duncan’s test for significant difference in data was used. The images were drawn using OriginPro 2016 (Origin Lab Corporation, Northampton, MA, USA), and fitted curves and regression analysis were performed in Figure 4 and Figure 6, Table 2, and Supplementary Figure S1.

## 5. Conclusions

This study investigates the effects of over-irrigation and water-deficit PRD on the diurnal variation of photosynthesis in alfalfa leaves, as well as its relationship with soil moisture. It also explores the reasons behind the increased WP of alfalfa resulting from over-irrigation. Our results suggest that PRD is not the main factor causing stomatal and non-stomatal limitation in alfalfa, but water stress is the main factor. Shallow soil plays an important role in regulating leaf photosynthesis. In addition, over-irrigation may increase the photosynthetic rate of alfalfa leaves, thereby increasing its yield and promoting its water productivity.

## Figures and Tables

**Table 1:** Partial root-drying subsurface drip irrigation for alfalfa in 2017.

Treatments	Degree of Water Deficit	Seedling Irrigation /mm	Irrigation Frequency /No.	Effective Rainfall /mm	Irrigation Quota/mm	Irrigation Time/(M-D)	Irrigation Volume/mm
PRD0	Severe water deficit	50 ^a^	5 ^b^	13.4	0	17 June, 24 June, 1 July, 8 July, 15 July	50
PRD1	Moderate water deficit (45% ET_0_)	50 ^a^	5 ^b^	13.4	10		100
PRD2	Full irrigaiton (89% ET_0_)	50 ^a^	5 ^b^	13.4	20		150
PRD3	Over-irrigation (134% ET_0_)	50 ^a^	5 ^b^	13.4	30		200

^a^ The number of sprinkler irrigations during the seedling stage was 5 times, and the irrigation quota was 10 mm; ^b^ The frequency of irrigation during the seedling stage was not included.

**Table 2:** Nonlinear regression coefficients and statistical parameters between shallow soil moisture and photosynthetic parameters.

Soil Depth/cm	Photosynthetic Parameters	Regression Coefficient			Coefficient of Determination
		y_0_	A	R_0_	*R* ^2^
10	*P_n_*/(μmol m^−2^ s^−1^)	36.6897	−715.0556	−0.4910	0.9864
	*T_r_*/(mmol m^−2^ s^−1^)	23.2425	−316.3945	−0.4158	0.8571
	*G_s_*/(mmol m^−2^ s^−1^)	0.6295	−3.7961	−0.2889	0.8462
20	*P_n_*/(μmol m^−2^ s^−1^)	38.5017	−2479.1211	−0.4939	0.8820
	*T_r_*/(mmol m^−2^ s^−1^)	24.6012	−1189.0500	−0.4453	0.6913
	*G_s_*/(mmol m^−2^ s^−1^)	0.6756	−10.2782	−0.3129	0.6951

*P_n_*: photosynthetic rate; *T_r_*: transpiration rate; *G_s_*: stomatal conductance; *C_i_*: intercellular CO_2_ concentration.

**Table 3:** Effect of PRD on alfalfa hay yield, water productivity, and irrigation water productivity.

Treatments	Hay Yield/(kg hm^−2^)	WP/(kg hm^−2^ mm^−1^)	IWP/(kg hm^−2^ mm^−1^)
PRD0	2752.27 ± 278.09 Dd	22.81 ± 2.29 Bb	55.05 ± 5.56 Aa
PRD1	3546.00 ± 221.22 Cc	20.88 ± 1.49 Bb	35.46 ± 2.21 Bb
PRD2	4356.80 ± 109.02 Bb	22.91 ± 0.64 Bb	29.05 ± 0.73 Bc
PRD3	5825.47 ± 275.44 Aa	30.50 ± 1.16 Aa	29.13 ± 1.38 Bc

WP: Water productivity; IWP: Irrigation water productivity. Capital letters indicate significant differences among treatments at the *p* < 0.01 level, and lowercase letters indicate significant differences among treatments at the *p* < 0.05 level.

## Data Availability

The data are contained within the article.

## Supplementary Materials

The following supporting information can be downloaded at https://www.mdpi.com/article/10.3390/plants14111573/s1. Figure S1: Relationship between intercellular CO_2_ concentration with net photosynthetic. Figure S2: Location of study sites (a), completely randomized block design (b), actual layout of partial root zone drying subsurface drip irrigation (c) and design of partial root zone drying subsurface drip irrigation (d).
