# Supplementary material for: Alfalfa Photosynthesis Under Partial Root-Zone Drying: Diurnal Patterns and Its Non-Stomatal Limitations
Source: Plants (Basel). 2025 May 22;14(11):1573. doi: 10.3390/plants14111573 (PMC12157153; doi:10.3390/plants14111573)
Supplement: Supplementary file 1 [file plants-14-01573-s001.zip › plants-3635146-supplementary.pdf]

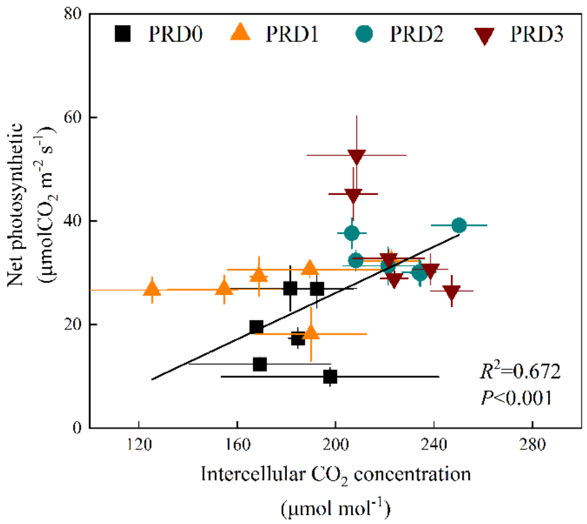

Supplementary Figure 1. Relationship between intercellular CO_2 concentration with net photosynthetic.

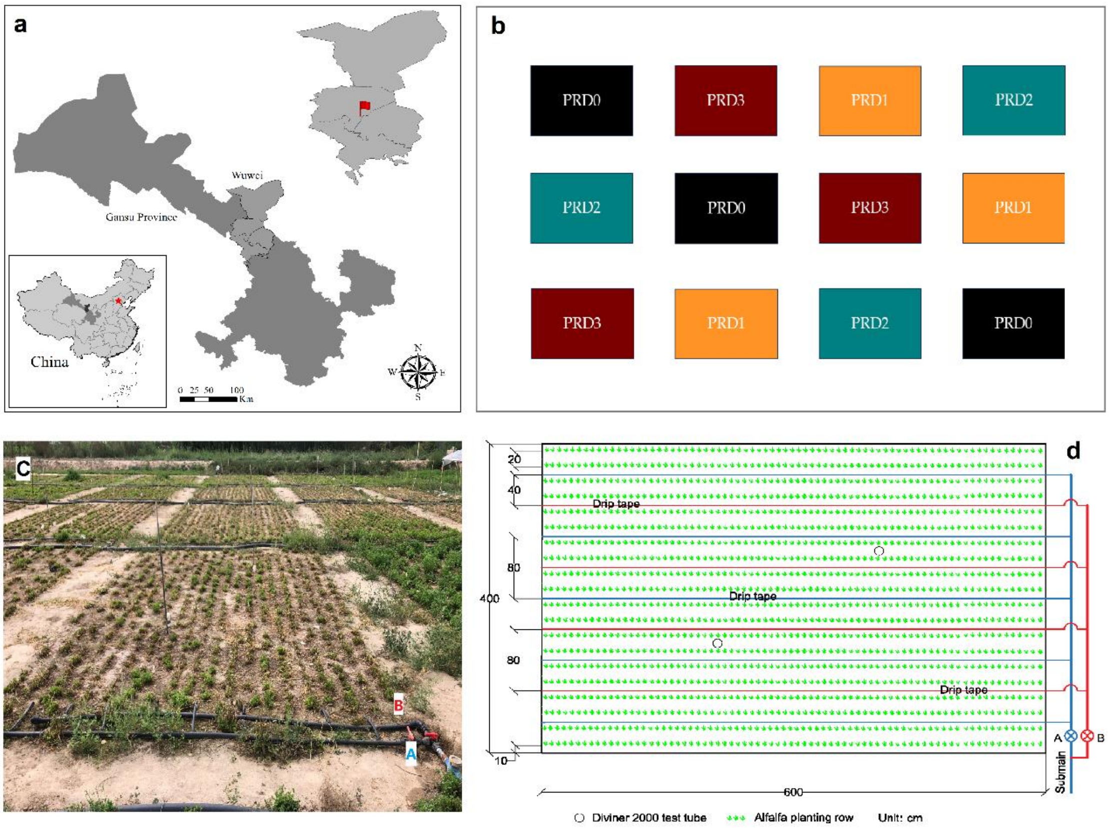

Supplementary Figure 2. Location of study sites (**a**), completely randomized block design (**b**), actual layout of partial root zone drying subsurface drip irrigation (**c**) and design of partial root zone drying subsurface drip irrigation (**d**).
